# Permanganate Oxidation of Microcystin-LA: Kinetics, Quantification, and Implications for Effective Drinking Water Treatment

**DOI:** 10.1155/2019/3231473

**Published:** 2019-05-28

**Authors:** David C. Szlag, Brian Spies, Regina G. Szlag, Judy A. Westrick

**Affiliations:** ^1^Department of Chemistry, Oakland University, Rochester, MI 48309, USA; ^2^Department of Chemistry, Wayne State University, Detroit, MI 48202, USA

## Abstract

Permanganate pretreatment of drinking water is effective in transforming dissolved, noxious contaminants and in reducing halogenated by-products. Permanganate targets specific compounds such as taste and odor compounds, disinfection precursors, manganese, and natural organic contaminants that are not removed readily by conventional treatment alone. Cyanobacterial blooms (cHABs) can increase disinfection by-product precursors as well as the cyanotoxin, microcystin (MC), a potent liver toxin. MC toxicity is conferred by a unique, conserved amino acid, Adda, that inhibits protein phosphatase 1 and 2A. Although over 150 MC congeners have been reported, thousands of MCs are statistically possible. Over the last ten years, one congener, MC-LA, has been reported with increasing frequency, making it one of the most common cyanotoxins identified in North American freshwater systems; yet its oxidation has not been widely studied. Frequently, Adda specific enzyme-linked immunosorbent assay (ELISA) and protein phosphatase inhibition assay (PPIA) are used to quantitate total MCs to evaluate treatment efficiency and exposure. Anecdotal reports suggest that MC degradation products can cause interference with the Adda-ELISA. MC-LA was used as the model MC compound in this study. PPIA quantitation of MC-LA in water agreed with liquid chromatography high resolution mass spectrometry (LC/HRMS), whereas the ELISA quantitation did not agree with LC/HRMS quantitation. We determined the second order rate constant for MC-LA as 118 ± 9 M^−1^ s^−1^, the activation energy to be 21.2 kJ mol^−1^, and the rate to be independent of pH between pH 6 and 9. Ten oxidation products (OPs) were observed by LC/HRMS and three primary reaction pathways are proposed. The reaction pathways were used to explain differences in the quantification by Adda-ELISA, HRMS, and PPIA. The oxohydroxylation of MC-LA produced two major OPs, C_46_H_67_N_7_O_14_ [M+H]^ +^ = 942.4819 and C_46_H_69_N_7_O_15_ [M+H]^+^ =960.4925. Major OPs may contain an unmodified Adda and are the likely cause of interference with the Adda-ELISA. Several governmental agencies recommend the use of the Adda-ELISA to determine the MC quantitation for treatment efficiency and customer exposure; yet our results suggest that these or other OPs interfere with the Adda-ELISA causing artificially high values for total MCs.

## 1. Introduction

The severity and frequency of cyanobacteria blooms are increasing due to a combination of factors including increased nitrogen and phosphorous pollution, climate change, and altered system ecology which in some cases may be the result of invasive species [1, 2]. Cyanobacteria blooms impact drinking water treatment efficiency by increasing chemical demand and particulate load, resulting in increased risk of pathogen and cyanotoxin breakthrough [3, 4]. Microcystins (MCs) are the most common class of cyanotoxins. During log-growth phase of the bloom, approximately 90% of the microcystin is intracellular and 10% is extracellular. Most treatment processes have specific targeted efficiencies for particulate and/or contaminant removal; to remove MCs, both types of processes must be optimized. Quantitative tools used to monitor MC drinking water treatment removal efficiency and exposure are critical for consumer safety. Ideally, these tools, i.e., assays, would determine the concentration of MCs as well as the toxicity of the water and would be simple enough to be performed at the treatment plant. Our goal was to vet the use of two commercial MC assays to quantitate MCs during permanganate oxidation and compare their results to the quantitation of MCs by LC/HRMS. Our hypothesis was that the quantitation by Adda-ELISA, PPIA, and LC/HRMS would agree.

**Figure 1:**
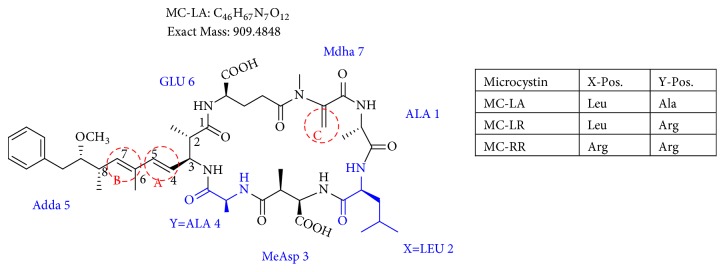
General structure of MC-LA showing variable amino acid positions and the primary (alkene) sites subject to permanganate oxidation.

MCs are cyclic heptapeptides of varying amino acid composition with respect to two positions (“X” and “Y”) that contain variable L-amino acids as shown in Figure 1. MCs are comprised of three D-amino acids: alanine (Ala), methylaspartic acid (MeAsp), and glutamic acid (Glu) (Figure 1). The structure also includes two unique amino acids: N-methyldehydroalanine (Mdha) and 3-amino-9-methoxy-2,6, 8-trimethyl-10-phenyldeca-4(E),6(E)-dienoic acid (Adda) (Figure 1). The amino acid Adda is found in position 5 of the peptide ring in all MCs, and it is this moiety that is responsible for the toxicity. The MCs inhibit irreversibly protein phosphatase 1 (PP1) and protein phosphatase 2A (PP2A) and act as hepatotoxins because they are preferentially transported into liver cells. The toxicity of the various congeners varies by two orders of magnitude with MC-LR and MC-LA noted as the most toxic [5]. MC-LA is prevalent in several regional water sources, but has not been reported historically as widely as MC-LR [6]. Not many comprehensive oxidative treatment studies have investigated the oxidation of MC-LA, even though it has been determined to have one of the slowest oxidation rates with the most widely used oxidant, chlorine [7].

The primary mode of human MC exposure is through drinking water. Historically, conventional water treatment facilities have been able to meet the World Health Organization (WHO) guidance value of 1.0 *µ*g/L MC-LR [8]. The US EPA has expanded monitoring from a single congener, MC-LR, to encompass all MC congeners referred to as total MCs. In 2015 US EPA released a two-tier drinking water health advisory (HA) based on age: preschool at 0.3 *µ*g/L total MCs, and above preschool age at 1.6 *µ*g/L total MCs [9]. Infant and toddlers ingest 5 times more liquid to body mass than school-age children and adults, hence, the decrease from 1.6 *µ*g/L for school-aged children and adults to 0.3 *µ*g/L total MCs for preschool-aged children. More countries will likely post similar guidance. Consequently, the drinking water industry is tasked to determine the total concentration of a class of compounds containing many congeners at the sub-part-per-billion concentration, a challenging task. Understanding the variability in oxidation kinetics of MCs and the oxidation pathways is needed, so that drinking water treatment can be optimized for MC removal and to minimize toxic OPs. Interferences must be identified and quantified to ensure accurate process monitoring.

Two classes of commercial biochemical assays, ELISA [10] and PPIA [11], have become standard technologies to quantitate total MCs for drinking water utilities. These kits may use different antibodies or enzymes, but generally respond to all MC congeners, albeit variably. Several governmental agencies recommend the use of the Adda-ELISA in the mistaken notion that Adda alone confers toxicity. To date, Adda-ELISA quantitation has not been directly correlated to MC toxicity. The PPIA method is based on the enzymes which the MCs inhibit, and its quantitative response has been correlated to toxicity. Some studies have observed that the total MC concentrations derived from ELISA and PPIA are often higher than the total MC concentration derived from LC/MS [12–14]. Specifically, after chemical oxidation treatment, the Adda-ELISA total MC concentration has been reported to be higher than that reported by LC/MS [15, 16]. There is a need to determine which OPs are causing a response with the Adda-ELISA or PPIA and what their relative concentrations are under different reaction conditions.

Often permanganate is added at the source water intake. Anoxic source water may contain metals in a reduced state, and the initial permanganate demand comes from their oxidation. Oxidative pretreatment of water using permanganate is employed commonly to oxidize nuisance natural contaminants such as taste and odor compounds. Permanganate will selectively oxidize alkenes, and arguably does not rupture cells and release MCs at normal treatment dosages and contact times as determined by LC/MS [17–19]. In the early literature, the basic permanganate mechanism was believed to be the production of vicinal dihydroxyl or carbonyl products [20]. More recent work by Kim et al. [21] describes the permanganate oxidation progressing through vicinal hydroxyl-carbonyl pathways more like ozone oxidation [22, 23] rather than vicinal dihydroxy OPs produced via chlorine oxidation [24]. Because of its selective oxidation, permanganate treatment may be useful early in the treatment process for degradation of MCs as part of a multibarrier oxidation process to achieve the new low concentration guidance for total MCs.

To date, three drinking water utilities in United States have posted “do not drink” advisories because of MCs detected in their finished drinking water. Carroll Township, OH (2013); Toledo, OH (2014); and Salem, OR (2018) reported MCs concentrations above 1.0 *µ*g/L total MC. Because they serve 400,000+ residents, the Toledo, OH, advisory was the most publicized. The finished drinking water was reported to be above 1.0 ug/L total MCs as measured by Adda-ELISA, but these results were not validated by LC/MS/MS. Since “do not drink” advisories result in financial loss for businesses and loss of consumer confidence in the local water authority, identifying potential interferences in analytical methods that result in false positives is as critical as identifying potentially toxic OPs.

Source water MC concentrations often exceed 1 *µ*g/L, and it is not unusual for concentrations to range from 5 to 20 *µ*g/L total MCs. Our research focuses on how permanganate can be used as an effective treatment barrier and on quantification of MCs after treatment. In order to determine permanganate efficiency, we determine how congener chemical variation impacts oxidation rates; how the cyanobacteria cell is impacted by permanganate oxidation; and how variations in source water quality (pH and natural organic compound) affect permanganate oxidation. On-site quantitation is essential for monitoring the treatment process. We evaluated the Adda-ELISA and PPIA on-site methods by comparing the purported rate constants and by determining the oxidation reaction mechanism and pathways by LC/HRMS. Furthermore, determining the oxidation pathway provides insight regarding residual toxicity of OPs.

## 2. Materials and Methods

### 2.1. Microcystins

To compare our procedures to other researchers, MC-LR, -LA, and -RR were purchased from Enzo Life Sciences, Inc. For the majority of the oxidation experiments, however, MC-LA was purified from a* Microcystis* bloom/scum harvested from Upper Klamath Lake, OR, in 2015. MCs were released from the cells by three freeze/thaw cycles. Lysate was centrifuged in a swinging bucket then vacuum filtered through a 47 mm 0.4 *µ*m glass fibre filter. A crude extract was made by passing the lysate through a 60 cm^3^ C18 solid phase extraction column (Thermo Fisher Scientific, USA), discarding the effluent, and making the crude extract by eluting the bound material with 100 % HPLC grade methanol. MC-LA was separated from the crude extract by high performance liquid chromatography with a photodiode array detector using a C18 semipreparative column (Atlantis dC18 OBD Prep Column, 100A 5*µ*m 10 mm x 100 mm column, Waters Corp.). MC-LA was separated based on retention time and by UV spectrum. The MC-LA was validated by high resolution mass spectrometry (HRMS). Concentrated MC stock solutions were prepared at 0.5 mg/ml in MS grade methanol. Beer's Law with a molar extinction coefficient of 39,800 L·mol^−1^·cm^−1^ was used to determine the actual concentration of the MC-LA stock solution prior to dilution for the batch experiments [25].

A four-place mini jar test apparatus was used for all laboratory experiments with the paddle mixer set at 50 rpm. A 200 mL sample of DI, buffered (ammonium carbonate), or lake water was dispensed into each reaction chamber of the mini-mixer. The jar test unit was placed in a temperature-controlled cabinet to obtain the desired temperature 30 minutes prior to the start of batch experiments to reach thermal equilibrium. An intermediate stock solution of MC was prepared in DI water from the standardized stock. The MC stock solution was pipetted into the reaction chamber to obtain an initial concentration of 50 *µ*g MC/L for kinetic tests. Prior to the start of the experiment, 10uL of 0.05M sodium thiosulfate was preloaded into 1.4mL conical microfuge tubes that would receive samples to minimize delays in quenching the reaction. Prior to the addition of permanganate, a control sample of 1.0 mL was taken from each chamber (t = 0 seconds). Stock permanganate solution was added to the reaction chamber to bring the concentration to the desired 2.0 mg/L MnO_4_^−^, and 1.0 mL aliquots were collected at specified time intervals into the tubes preloaded with sodium thiosulfate. Generally, the kinetic studies of MC-LA were performed at six time points: 0, 2, 4, 6, 20, and 40 minutes. Temperatures for the Arrhenius plot were 4, 10, 18, 25, 30, and 35°C. Two temperatures, 25 and 35°C, were run for the experiments in which all three methods, Adda-ELISA, PPIA, and LC/HRMS, were used to quantitate MCs. These samples were immediately capped and vortexed for 6-10 seconds. Samples were held on ice for the duration of the batch experiment, then stored at 4°C, and analyzed within 48 hours. Prior to analysis by LC/TOF, PPIA, or ELISA, samples were centrifuged in a microfuge to pellet MnO_2_.

The sodium thiosulfate quenching solution was prepared by dissolving 3.01g of sodium thiosulfate in 25mL of 18 M-*Ώ* deionized water. This stock solution was stored in an airtight, amber bottle away from light. The stock solution was diluted 1:10 as needed for experiments.

The enzyme-linked immunosorbent assays (ELISA) were purchased from Abraxis, Inc. Calibration curves for MCs were quantitated using the response curve of MC-LR using standards: 0, 0.15, 0.5, 1.0, 2.5, and 5.0 *µ*g/L. All samples were diluted 20-fold to bring them into the range of the standard curve. All samples and standards were run in duplicate. Plates were quantified using a BioTek Synergy H1M microplate reader. A four-parameter curve fit was performed using BioTek Gen5 software.

Protein Phosphatase Inhibition Assay (PPIA) was developed by Zeulab S.L. Zaragoza, Spain, and distributed in the USA by Abraxis, Inc. Calibration curves for MCs range from 0.25 – 2.5 *µ*g/L. All samples were diluted 20-fold to bring them within the range of the standard curve. This test is based on inhibition of protein phosphatase 2A. All samples and standards were run in duplicate. Calibration curves for MCs were quantitated using the response curve of MC-LR. A four-parameter curve fit was performed using the BioTek Gen5 software.

LC/MS analysis was performed by HPLC (Agilent 1200) with a ZORBAX Eclipse Plus C18 (i.d. 2.1 × 50 mm, 1.8 mm) using gradient (10-100%) conditions with 0.1% formic acid and acetonitrile (flow rate: 0.4 mL min^− 1^). An Agilent 6520 quadrupole-time-of-flight (Q-TOF) was used for identification and quantitation. MS conditions were as follows: ionization, ESI; nebulizer, 50 psi(N_2_); drying gas, 10 L min^− 1^, 350°C (N_2_); Vcap, 5000 V; fragmentor, 100 V; scan,* m/z* 100–1500; 10,000 transients/scan, 10,000; references,* m/z* 121.0509 and 922.0098; and collision energy, 15–30 eV.

### 2.2. Pseudo-First-Order Kinetic Analysis

The oxidation reaction rate of MC-LA by MnO_4_^−^ can be expressed as

(1) $$\begin{array}{c} r=k{\left[\;MC\text{-}LA\;\right]}^{x}{\left[\;{{MnO}_{4}}^{-}\;\right]}^{y} \end{array}$$

where r is the reaction rate, k is the rate constant, [MC-LA] and [MnO_4_^−^] are concentrations, and x and y are the reaction orders with respect to MC-LA and MnO_4_^−^, respectively [28]. When the concentration of MnO_4_^−^ is excessive, (1) can be simplified to (2) and (3):

(2) $$\begin{array}{c} r=k^{\prime }{\left[\;MC\;\right]}^{x} \end{array}$$

where the product of the 2nd order rate constant and permanganate concentration is effectively constant and k′ is known as a pseudo-first-order rate constant.

(3) $$\begin{array}{c} k^{\prime }=k{\left[\;{{MnO}_{4}}^{-}\;\right]}^{y} \end{array}$$

This allows the use of the first-order integrated rate law and the determination of a pseudo-first-order rate constant k′.

(4) $$\begin{array}{c} \ln\left(\;\frac{MC}{MCo}\;\right)=-k^{\prime }t \end{array}$$

The natural log of the ratio, MC concentration divided by the initial MC concentration, was plotted versus time. The slope of this line is equal to the pseudo-first-order rate constant. The concentration of permanganate is present in 20-fold excess relative to the MC-LA and its concentration is effectively unchanged over the 40-minute experiment. The second order rate constant is calculated by dividing k′ by the molar concentration of permanganate.

## 3. Results and Discussion

### 3.1. Oxidation Kinetics of MC-LA with Permanganate

Chemical oxidation is considered an excellent drinking water treatment barrier for MC, such that America Water Work Association has a web-based modeling program to assist drinking water management to estimate the efficiencies of oxidation removal by chlorine, permanganate, and ozone [29]. This program uses published kinetics, pH dependent kinetics, and activation energies (temperature dependent kinetics) in their predictive empirical model. The authors of this tool have identified four reoccurring problems: (1) only a limited number of MCs have been investigated; (2) often temperature and pH dependent experiments are not performed; (3) the impact of NOM and cyanobacteria cells on oxidation kinetics is highly variable; and (4) kinetics determined by LC/MS are different from kinetics determined by Adda-ELISA. Our investigation provided temperature and pH dependence data on a less examined MC (MC-LA). By using Adda-ELISA, PPIA, and LC/HRMS to measure MC concentrations, we are able to show that OPs were likely binding to the Adda-ELISA antibody. Finally, elucidation of the reaction mechanism by LC/HRMS gives insight as to which potential OP products interfere with Adda-ELISA.

**Figure 2:**
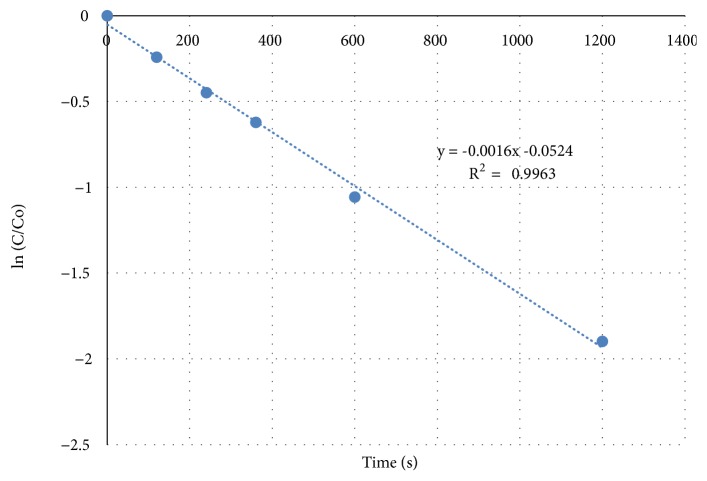
Example MC-LA pseudo-first-order oxidation with 2.0 ppm permanganate.

We observed second order rate constants 120 ± 9 M^−1^· s^−1^ and 114 ± 8 M^−1^· s^−1^ for our purified and commercially available MC-LA, respectively, with 2.0 ppm MnO_4_^−^, at pH 6.8, and 18 ± 1°C. Combining experiments (N=9) yielded an average second order rate constant at these conditions of 118 ± 9 M^−1^· s^−1^. This is approximately 25% lower than previously reported values by Kim et al. [21] and Ding et al. [26]. We also conducted a limited number of experiments with MC-RR and MC-LR. Our rate constant values for MC-LR and MC-RR are consistent and in the range observed by other researchers and follow the trend MC-RR > MC-LR > MC-LA (Table 1). Correcting for the temperature difference between our conditions and those reported by Kim et al. [21] or Ding et al. [26] would increase our values by eight percent. Sample data are shown in Figure 2.

**Figure 3:**
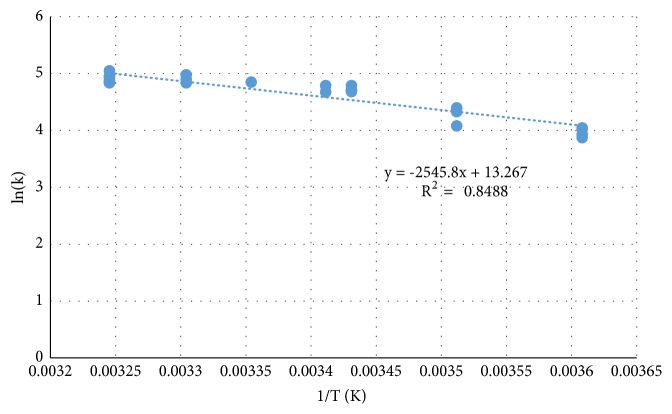
Arrhenius plot for MC-LA over the temperature range 4°C to 35°C.

The activation energy (E_a_) was determined by taking the natural log of the Arrhenius equation and plotting ln k versus 1/T (Figure 3). The coefficient of determination (R^2^) for the values in this study (N=24) over the temperature range 4°C to 35°C was 0.85. The E_a_ was 21.2 ± 1.9 kJ mol^−1^, pH was 6.7 ± 0.2, and the preexponential factor was 577,962 M^−1^ s^−1^. This E_a_ value for MC-LA is comparable to the value 21.5 ± 0.6 kJ mol^−1^ reported by Kim et al. [21].

**Figure 4:**
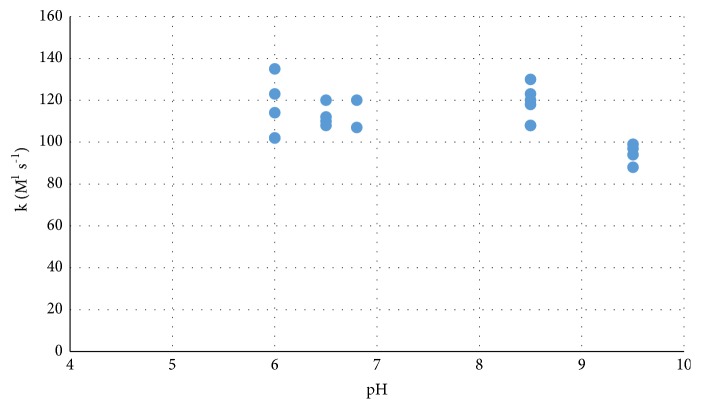
Variation of the MC-LA second order rate constant from pH 6 to 9.5.

We investigated the effect of pH on the rate constant over the range from pH 6 to pH 9.5 (Figure 4) and found that pH has a negligible effect on k between pH 6 and 8.5 but appears to drop off at pH 9.5. Often pH of the water during a bloom is > 8.5, and the reduction of oxidation rate will reduce treatment efficiency.

Many groups have studied the effects of NOM and the type of NOM that reacts with permanganate. Jeong et al. [21] show that fulvic and humic acids react with permanganate, while polysaccharides and proteins are much less reactive. The effect of Lake Erie water (Table 2) was evaluated on the reaction of permanganate and MC-LA. Within experimental error there was no effect on the rate constant at 22°C and 35°C. We did not completely characterize the NOM in the lake water, but the Lake Erie water is low in humic and fulvic acids and higher in proteinaceous NOM [30]. Permanganate reacts more slowly with proteins or amino acids relative to alkenes.

We further support this observation with experiments containing* Microcystis* cells (*M. viridis*) as shown in Table 3. There is no statistical change in the k value as the amount of cyanobacteria cells is increased. Furthermore, there is no reduction in k when a cell lysate was added. More studies of this phenomenon with wild* Microcystis* strains should be performed before general conclusions are made, however. The* M. viridis *cells used in this experiment have been grown in culture for many years and do not exhibit colonial morphology; they primarily grow as single or double cells without a mucilaginous layer that is often observed in wild strains.

In general our observations are consistent with previous works that indicates that permanganate has a low reactivity with proteinaceous material and that, at moderate dosages, permanganate is unlikely to lyse cyanobacteria cells.

### 3.2. Comparison between the Quantitation and Kinetics by Adda-ELISA, PPIA, and LC/HRMS

Both the Adda-ELISA and PPIA can use a microplate reader format providing drinking water utilities a readily available on-site platform versus the LC/HRMS which is a costly instrument that needs a highly trained technician. Several natural water studies have compared the results from these three technologies [12–14] showing general agreement, but noting that the Adda-ELISA often reports higher concentrations than LC/MS, whereas the PPIA reports lower concentrations than the LC/MS. Limited studies have investigated Adda-ELISA and PPIA MC quantitation of waters that have undergone chemical oxidation with chlorine, ozone, and chloramines [15, 16]. These results suggest that OPs from chlorination, ozone, and chloramines react with the Adda-ELISA antibody and the PPIA.

This work determined the apparent second order kinetics of the permanganate oxidation of MC-LA by Adda-ELISA, PPIA, and LC/HRMS (Table 4) at 25± 1 C and 35.5 ± 1 C. Since buffered DI water was used for this study, the permanganate demand is based on MC-LA and only MC-LA OP products are present. The k_PPIA_ and k_MS_ at both temperatures are very similar, while the k_ELISA_ has decreased by about 40%. These data suggest that the MC-LA OPs from permanganate oxidation may be binding with the Adda-ELISA, but not the PPIA.

The permanganate OP pathways for MC-LR have been elucidated by Kim [21]. The primary paths were (1) oxidation at one of the three alkenes to make a hydroxyl ketone and (2) oxidative cleavage at the Adda diene or Mdha. OPs that may bind with Adda-ELISA include the oxidation products of the Mdha because the Adda remains intact and the oxidative cleavage of the C4-C5 alkene. This cleavage leaves the ring intact and produces (4S,5S,E)-5-methoxy-2,4-dimethyl-6-phenylhex-2-enal (MMPHA) and/or the (4S,5S,E)-5-methoxy-2,4-dimethyl-6-phenylhex-2-enoic acid (MMPHH). MMPHH and MMPHA chemical structures are like Adda and may interfere with the Adda-ELISA.

**Figure 5:**
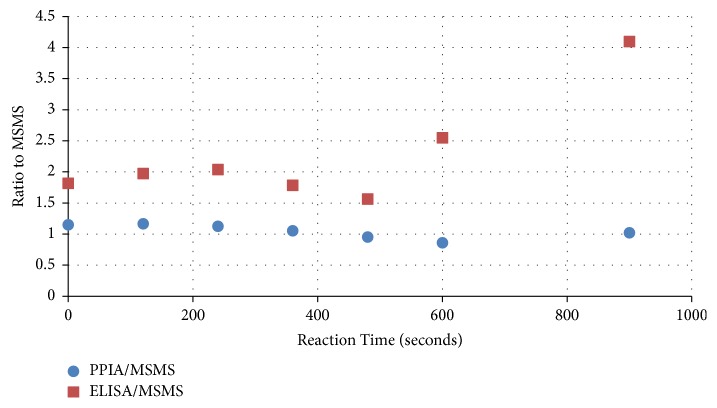
PPIA and Adda-ELISA normalized by LC/HRMS show that the ratio of the response of the Adda-ELISA increases as the reaction progresses.

The Adda-ELISA and PPIA results can be normalized by the corresponding LC/HRMS concentration (Figure 5). Similar to the observations reported by Guo et al. [15], the Adda-ELISA concentrations from time 0 to 480 seconds were about twice the concentration determined by the LC/HRMS. This is becuase MC-LA binds more efficiently to the antibody than MC-LR for which the calibration curve is generated. The time 0-minute sample is taken before the addition of permanganate for each experiment and diluted 20x (as are all samples) and serves as a control for each experimental run. The starting concentration of each experiment is 50 *µ*g/L MC-LA and is determined independently by measurement of the MC-LA stock solution using UV spectroscopy. After 6-10 minutes, the MC-LA concentration determined by the Adda-ELISA is considerably larger than the LC/HRMS. The ratio between Adda-ELISA and LC/HRMS increased from 2 to 4. This change in ratio reflects the change in the second order rate kinetics from concentrations determined by Adda-ELISA at 61 M^−1^sec^−1^ versus the 100 M^−1^sec^−1^ determined by LC/HRMS. These data suggest that, after 480 seconds, an OP(s) is at a high enough concentration to interfere with the Adda-ELISA. It is reasonable to suspect that the OP(s) has an intact Adda, MMPHA, or MMPHH.

The normalized PPIA results are close to 1.0 across the entire time interval. Often, researchers use the PPIA to predict MC toxicity. The MC concentrations and kinetic rate determined by PPIA matched the concentrations and kinetic rate determined by LC/HRMS (Table 4) within experimental error. The OPs formed do not appear to inhibit PP2A suggesting the OPs are unlikely to be toxic. Both the PP2A and Adda-ELISA bind to the Adda group in the MC, but they appear to react differently to the MC-LA OPs suggesting that the OPs reacting with Adda-ELISA do not inhibit protein phosphatase. These results suggest that, after permanganate oxidation, OPs can result in an Adda-ELISA false positive that may not be related to the toxicity of the water. It should be noted that the hepatotoxicity is also a function of the transport of MCs by the organic anion transporter (cell transmembrane protein) into liver cells. Consequently, future studies of OPs should include the use of cell-based assays to determine if OPs that do inhibit PP1 and PP2A are capable of entering liver cells and exhibiting toxicity.

### 3.3. Oxidation Products and Pathways

Over the last couple of decades, scientists have used mass spectrometry to determine the oxidation reaction pathways of MC-LR for a variety of oxidants. Understanding the pathway provides insight into the detoxification of MCs. Since the Adda group is the toxic center of the MC, the oxidation of Adda is critical in the detoxification/inactivation of MCs. However, biodegradation studies have shown that ring opening is another mechanism of detoxification [31, 32]. Other studies have suggested that the kinetics determined by mass spectrometry (as shown above) do not provide the same kinetics as the Adda-ELISA [15, 16], yet no mechanism was provided to explain the disagreement. The most likely explanation is that the Adda remains intact after oxidation and binds to the antibody; it is also possible that the antibody is not that specific for Adda, and a piece(s) of the MC OP cross-reacts with the Adda antibody. Elucidation of the oxidation pathway(s) can provide insight as to the source of the false positives and promote design of better monitoring strategies.

Three previous studies proposed oxidation pathways for MC-LR with permanganate [20, 21, 26]. No previous study has elucidated the pathway for MC-LA. In the two earlier studies, dihydroxylation of the Adda and Mdha alkenes was the proposed product pathway. Dihydroxylation across the alkene is the permanganate alkaline (base) reaction frequently used by synthetic chemists. Jeong et al. [20] also suggested hydroxylation of the Adda benzene. Kim et al. [21] discounted these pathways as they did not detect the dihydroxy MC-LR intermediate products, nor did they think it is likely that permanganate would form hydroxylated benzene OPs given extremely slow rates of reaction for benzylic compounds and permanganate. Their pathway consists of 17 products formed via five reactions: (1) alkene to hydroxyketone; (2) alkene to aldehydes; (3) aldehyde to carboxylic acid; (4) amide to amine; or (5) carboxylic acid. They identified three classes of primary products: (1) hydroxyketone across one of the alkenes; (2) oxidation cleavage to form aldehydes; and/or (3) ketones. Secondary and tertiary oxidation proceeds via transformation of aldehydes to carboxylic acids, and hydrolysis of amide peptide bonds to an amine and carboxylic acid which opens the ring. Furthermore, their results implied that the *α*,*β* unsaturated carbonyl was less reactive than the diene to permanganate oxidation.

In our pathway studies, 1000 *µ*g/L MC-LA rather than 50 *µ*g/L MC-LA was used with 2.0 mg/L permanganate to assure generation of sufficient concentration of minor OPs that could be detected by LC/HRMS. We also extended the reaction time from 40 to 60 minutes.

We identified eight major and several minor OPs of MC-LA that are analogous to those identified in Kim et al.'s [21] work on MC-LR using similar LC/HRMS analysis. Our proposed oxidation pathways are analogous to the scheme developed by Kim et al. [21] for MC-LR oxidation with permanganate. Our labelling scheme follows theirs, albeit with the analogous formulas and masses representing MC-LA OPs. The OP's chemical formula, mass, label, retention time, mass difference, and proposed structure are reported in Table 5. Three major OPs, i.e., one site of oxidation, were formed within 2 minutes: the oxidation of the Adda diene (Figure 1) to form hydroxy ketones and the oxidative cleavage of the C4-C5 alkene to form an aldehyde product. Since the three products appear at the first-time point (2 min), it is not known if these OPs are produced in parallel or in series. These primary oxidation products undergo further oxidation. The oxidation reactions at the Mdha *α*,*β* unsaturated carbonyl seem to occur after the Adda diene reacts because they appear only at longer times.

**Figure 6:**
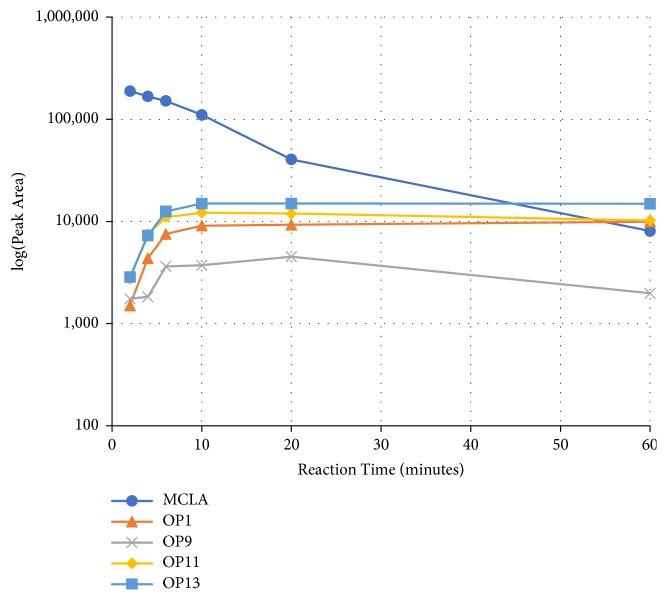
MC-LA major oxidation products formed via reaction with 2.0 ppm permanganate at 22°C.

The relative abundance of primary OPs from our permanganate MC-LA oxidation experiments is shown in Table 6 and Figure 6. The primary oxidation products we observed for the reaction of MC-LA with permanganate were generally analogous to those observed by Kim et al. [21] except for the aldehyde OP oxidized to the carboxylic acid. We observed MC-LA analogs to OP11, hydroxyketones forming at sites A or B (Figure 1), and analogs for OP1 and OP9 forming by oxidative cleavage at sites A and C, respectively. Products OP1 and OP11 continued to increase in concentration over the course of the one-hour reaction, whereas OP9 peaked at 20 minutes and began to decline by 60 minutes. We did not observe dihydroxy MC-LA (C_46_H_69_N_7_O_14_; [M+H]^+^ = 944.49747) or the aldehyde formed by oxidative cleavage at site B. This study, using MC-LA and all previous pathway studies with MC-LR, agree that OP1 analogs form rapidly and are the major product. Furthermore, this aldehyde OP should be nontoxic and is unlikely to react with the Adda-ELISA or the PPIA, given that the Adda is cleaved.

Secondary OPs, OP10 and OP13, appear at two minutes and OP13 is the major OP of MC-LA. Both OPs are formed by hydrolysis of an amide bond. If the HRMS response is relatively constant across the products, OP13 is the most abundant OP produced over the course of our one-hour reaction time. Kim et al. [21] observed the same behavior. An interesting deviation from Kim et al.'s observations is that we do not observe OP3, OP4, and OP7 within one hour [21]. The secondary OPs react via amide hydrolysis and result in the formation of carboxylic acids and opening of the peptide ring. In the Kim et al.'s study [21], OP7 (formed via oxidation of the aldehyde from precursor OP3 or OP6) was a major product along with primary OP1 and OP11. The minor products in Kim et al.'s study were analogous to OP3, OP4, OP9, OP10, and OP17 [21]. Similarly, we did not observe OP3, OP4, and OP17 while we did see OP5 and OP7. Our minor products were OP2, OP8, OP10, OP12, and OP15. OP16 and OP8 can be referred to as a tertiary OPs because they have been oxidized twice and hydrolyzed once. Oxidative cleavage to carbonyls and oxidation to hydroxyketone at the three alkenes are the first reactions to take place. We can infer that hydrolysis of the amide peptide bond is fast only after the initial oxidation of the alkene.

**Figure 7:**
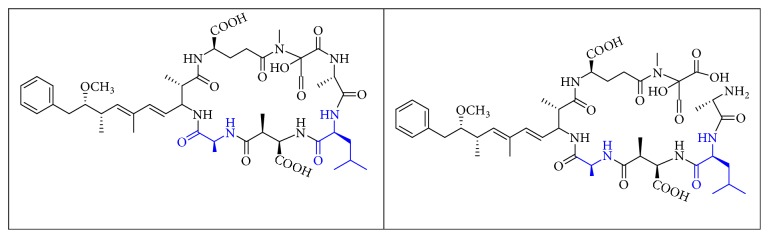
Two OP isomers, OP11 and OP13, with intact Adda that are likely interferents. Note that OP13 is an open ring.

From these observations we can infer that the major pathways for MC-LA transformation are MC-LA→OP11→OP13→P16, MC-LA→OP1=>P6, and MC-LA→OP9=> OP12. Many OPs have several possible structural isomers as well as stereoisomers. OP9 and OP10 have intact Adda and are likely to be toxic or interfere with the Adda-ELISA kit. They are not the major products, however, for MC-LA. OP9 is produced in relatively modest amounts relative to OP1, OP11, and OP13 but is not the likely interferent with the Adda-ELISA because it is converted to OP12. OP10 is likely to interfere with Adda-ELISA but is produced in minor amounts. Preliminary fragmentation studies confirm that P10 has an intact Adda. OP11 and OP13 have four possible isomers. OP11 and OP13 (Figure 7) with intact Adda are likely to interact with the Adda-ELISA. OP13 is likely to exhibit interaction with the Adda-ELISA but, because of the open ring, will not inhibit PP1 or PP2a. This is the most likely candidate for the interference with the Adda-ELISA. Subsequent work will attempt to isolate OP11 and P13 and test for interference with the Adda-ELISA and toxicity.

## 4. Conclusions

The results presented provide a better understanding of how permanganate reduces MC toxicity and identifies several practical implications. Chemical oxidation of MC with 2.0 mg/L permanganate with sufficient contact times can be an effective first barrier for the more hydrophilic MCs, MC-RR, and MC-LR that oxidize rapidly. We determined that MC-LA has much slower kinetics than previously reported. Regional difference in bloom ecology gives rise to different congener profiles. Identification of blooms dominated by MC-LA is essential to manage the treatment process. We tested permanganate oxidation with Lake Erie water and different levels of cells. The NOM and cells did not change the rate constants and confirmed the selectivity of the permanganate. Furthermore, there was no evidence that permanganate lysed the cells at 2.0 mg/L. We also showed that OP can interfere with the Adda-ELISA whereas the PPIA kit tracks the HRMS well. Furthermore, inhibition of PP2A is significantly reduced, implying toxicity is also reduced. The most likely OP for interfering with the Adda-ELISA kit is OP13, an open ring OP with intact Adda or the MMPHA/MMPHH. We also show that MC-LR and MC-LA congeners give rise to different ratios of analogous OPs and that further research is necessary regarding the toxicity of individual OPs. One significant area of concern is the effect of higher pH. Most cHAB events cause the pH of the source water to rise and it is not uncommon to observe source water with pHs between 8 and 9.5. It is possible that pHs at the higher range may slow the reaction and produce a different set of OPs that could enhance interference and toxicity.

## Figures and Tables

**Table 1:** Summary of second order rate constants for MCs.

MC-LA	MC-LR	MC-RR	Temp.	pH	Reference
k (M^−1^ s^−1^)	k (M^−1^ s^−1^)	k (M^−1^ s^−1^)	°C		

118 ± 9	320 ± 20	467 (N=1)	18 ± 1	6.8 ± 0.2	This study

160 ± 1.3	386 ± 20	520 ± 9	21 ± 1	7.2	Kim et al. [21]

170	408		23 ± 1	7.6	Ding et al. [26]

	290		23 ± 1	7	Jeong et al. [20]

	357 ± 18	418	20	7.2	Rodríguez et al. [27]

		469 ± 37	25	6.7	Chen et al. [28]

**Table 2:** Effect of natural lake water on the MC-LA second order rate constant.

Source	22°C	35°C
Filtered Lake Water	118 ± 15 M^−1^sec^−1^	144.5 M^−1^sec^−1^

DI	113.5 ± 6.5 M^−1^sec^−1^	138.5 ± 12.5 M^−1^sec^−1^

**Table 3:** The variation of the MC-LA second order rate constant in the presence of *M. viridis* cells.

0 cells/mL	31,000 cells/mL	160,000 cells/mL	160,000 lysed
114.5 M^−1^sec^−1^	122 M^−1^sec^−1^	114.3 M^−1^sec^−1^	128 M^−1^sec^−1^

**Table 4:** Apparent rate constants determined by three methods at 25°C and 35°C.

Initial Concentration Co (*µ*g/L)	Permanganate Concentration (ppm)	Temperature °C	k_PPIA_ M^−1^sec^−1^	k_MS_ M^−1^sec^−1^	k_ELISA_ M^−1^sec^−1^
50 MC-LA	2.0	25 ± 1	116 ± 4	100 ± 1.4	61.5 ± 5

50 MC-LA	2.0	35 ± 1	145 ± 1.4	134.5 ± 10	82 ± 25

**Table 5:** Description of MC-LA permanganate OP.

Description	OP	[M+H]^+^	OP Formula	RT	∆ (ppm)
Figure 1: MC-LA		910.4921	C_46_H_67_N_7_O_12_	10.4	-2.2

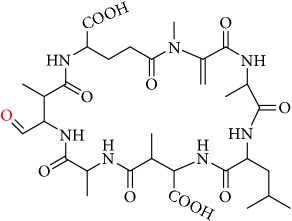	1	710.3356	C_31_H_47_N_7_O_12_	8.95	0.5

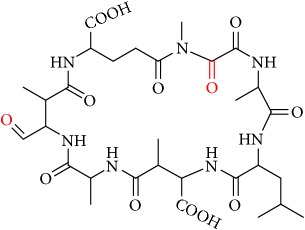	2	712.3154	C_30_H_45_N_7_O_13_	na	na

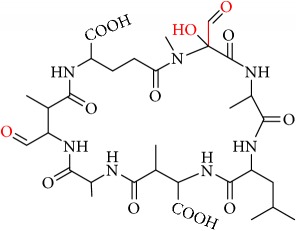	6	742.3254	C_31_H_47_N_7_O_14_	0.57	-1.8

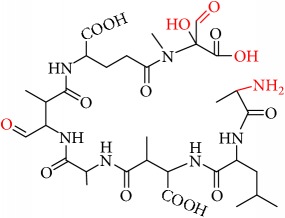	8	760.3360	C_31_H_49_N_7_O_15_	0.55	3.35

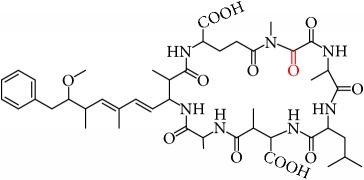	9	912.4714	C_45_H_65_N_7_O_13_	9.89	-2.17

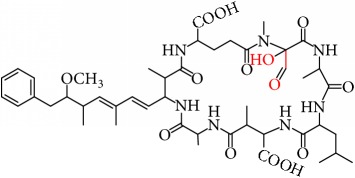	11	942.4819	C_46_H_67_N_7_O_14_	9.61	1.73

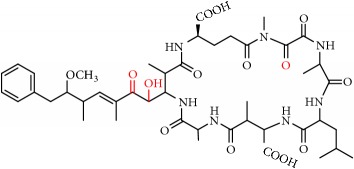	12	944.4612	C_45_H_66_N_7_O_15_	9.11	3.21

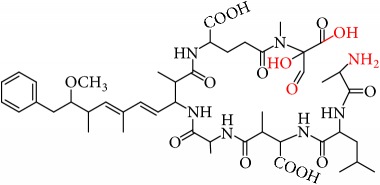	13	960.4925	C_46_H_69_N_7_O_15_	8.93	-0.2

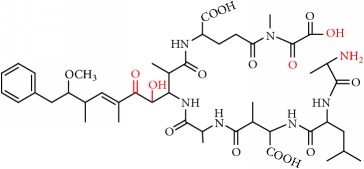	14	962.4718	C_45_H_67_N_7_O_16_	8.95	9.8

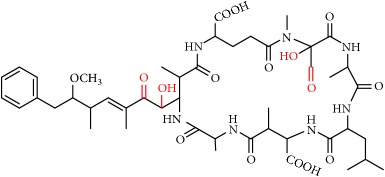	15	974.4718	C_46_H_67_N_7_O_16_	8.90	-0.37

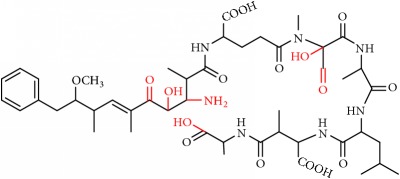	16	992.4823	C_46_H_69_N_7_O_17_	8.15	-1.53

**Table 6:** Proposed MC-LA OP analogous to the 17 MC-LR OP identified by Kim et al. [21]. Only 12 of the 17 products were identified in our studies with MC-LA.

	MCLA	OP1	OP2	OP6	OP8	OP9	OP10	OP11	OP12	OP13	OP14	OP15	OP16
M/Z	910.4921	710.3356	712.3149	742.3254	760.336	912.4714	930.4819	942.4819	944.4612	960.4925	962.4718	974.4718	992.4823

Time (m)	Area												

2	189187	1504	0	0	0	1750	444	2746	0	2854	0	0	0

4	167826	4368	0	0	0	1834	100	7539	0	7296	0	0	0

6	151568	7547	0	0	0	3629	213	11012	0	12552	0	0	0

10	110928	9106	0	0	0	3723	206	12217	0	14983	0	0	0

20	40476	9296	0	0	0	4527	100	11981	0	15009	314	0	0

60	8064	9951	581	2804	1309	1980	0	10257	891	14905	2028	1559	6939

## Data Availability

The data used to support the findings of this study are available from the corresponding author upon request.
